# Vaginal Versus Sublingual Misoprostol for Labor Induction at Term and Post Term: a Randomized Prospective Study

**Published:** 2014

**Authors:** Sedigheh Ayati, Fatemeh Vahidroodsari, Farnoosh Farshidi, Masoud Shahabian, Monavar Afzal Aghaee

**Affiliations:** a *Women*᾽*s Health Research Center, Ghaem Hospital, Mashhad University of Medical Sciences, Mashhad, Iran. *; b *Obstetrics Gynecologist, Mashhad University of Medical Sciences, Mashhad, Iran. *; c *Mashhad University of Medical Sciences, Cardio-Vascular Research Center, Mashhad, Iran. *; d *Department of Medicosocial, Biostatics Unit, Mashhad University of Medical Sciences, Mashhad, Iran.*

**Keywords:** Induction of labor, Misoprostol, Vaginal, Sublingual

## Abstract

We want to compare the efficacy and safety of vaginal versus sublingual misoprostol for cervical ripening and induction of labor. This randomized clinical trial was performed on 140 women with medical or obstetric indications for labor induction. The patients were randomly divided into two groups: vaginal and sublingual administration of misoprostol. In first group, 25 µg misoprostol was placed in the posterior fornix of the vagina and second group received 25 µg misoprostol sublingually, every 6 hours for 24 h. Maternal and neonatal outcomes were analyzed. There was no significant difference in the demographic characteristics between two groups. The main indication for cesarean section in both groups was fetal distress, followed by absence of active labor progress. Evaluation of cesarean indication was not significantly different in two groups; including fetal distress, absence of active labor, uterine over activity and failure to progress. The maternal complication in sublingual group included residual placenta (2%), tachysystole (2%), vomiting (12%), atoni (3.3%) and abdominal pain (5.5%), although there was no significant difference between two groups. Sublingual misoprostol is as effective as vaginal misoprostol for induction of labor at term. However, sublingual misoprostol has the advantage of easy administration and may be more suitable than vaginal misoprostol.

## Introduction

 Induction of labor is usually performed when the risks of continuing pregnancy are higher than the benefits of delivery (1-3). Undoubtedly, uterine cervical tissue ripening or its softening has a close relationship with success rate of delivery. 

There are several effective methods for cervix ripening including mechanical with osmotic dilators (4) or balloon catheters (5), and biochemical with prostaglandins (6), antiprogestins (7), or nitric oxide donors (8). Among many proper methods for cervical ripening, there is still no agreement on which method is the best for labor induction of cases with unripe cervix. 

Among prostaglandins, Misoprostol (PGE1) is widely used for «induction of labor» and cervical ripening (9-11). Cervical ripening occurs by the activation of prostaglandin E2 receptor. 

The most effective therapy for peptic ulcer is still unknown and prompts people to make great efforts to find better and more modern natural or synthetic agents (12). The prostaglandin E1 analogue (Misoprostol), synthetic 15 deoxxy-16 hydroxy-16-methyl is administrated for peptic ulcers therapy (9) (suppressed prostaglandin) used for cervical ripening and labor induction as well. Due to finding the Misoprostol, a relatively cheap and stable substance at room temperature without need to refrigeration for storage, like the other prostaglandins, with good proven efficacies, recently it is frequently used in obstetrics and gynecology for termination of pregnancy, especially at third trimester (13). However, some major complications are reported after administration of high doses of misoprostol, likewise uterine hyperstimulation and rupture (14). Vaginal and sublingual misoprostol have a rapid onset action, due to their prolonged activity and bioavailability (11). A sublingual dose of 50 mg every 4 h in most of cases, induce vaginal delivery within 24 hours and compared to an equivalent oral dose, less oxytocin augmentation is required (15,16). However, the previous studies found few significant differences among the effectiveness of different doses of the Misoprostol, oral, vaginal or sublingual (17). So this study is performed to compare the efficacy and safety of vaginal versus sublingual misoprostol with four hours interval for six doses for cervical ripening and induction of labor.

## Experimental

This randomized clinical trial was conducted on 140 pregnant women who were admitted at Department of Obstetrics, Mashhad University of Medical Sciences during September 2007 to March 2008. 

Inclusion criteria were gestational age>37 weeks on the basis of last menstrual period (LMP) or ultra-sonography at first trimester, need to pregnancy termination due to fetal or maternal indication, Bishop score < 7, gestational diabetes mellitus, singleton pregnancy, reassuring fetal heart rate tracing, cephalic presentation, low-located placenta, and mild preeclampsia.

Any cases of hypersensitivity to prostaglandin, temperature > 38 °C, previous cesarean delivery or other uterine surgery, placenta previa, chorioamnionitis, vaginal bleeding, fetal distress, need to immediate delivery, macrosomy, and polyhydroamnios were excluded from the study. 

The women were randomly divided into two groups: 50 cases in vaginal misoprostol group (group 1) and 90 cases in sublingual misoprostol group (group 2). The sample size was calculated with NCSS software (Power = 80%, Alpha = 0.05). The method of randomization was simple randomization. At first, all conditions of the study were completely explained for the women; if written informed consent was obtained, they were entered to the study. This study was approved by the Ethics Committee of Mashhad University of Medical Sciences. 

After randomization, for women in group 1, 25 µg misoprostol was placed in the posterior fornix of the vagina; and women in group 2 received 25 µg misoprostol sublingually. In each group, if needed, prescription was repeated up to 6 doses every 4 hours and vaginal examination was performed every 4 hours (18); if uterine contractions did not begin, the patient received another dose. In the presence of spontaneous and frequent contractions (duration of at least 40-50 seconds every 3 min), the next dose was not administered. If the patient did not enter into active labor (active labor: cervical dilatation of 3 to 5 cm or more in the presence of uterine contractions; latent phase: when the mother perceives regular contractions and usually ends at between 3 and 5 cm of dilatation (19)) 4-h after the last dose of misoprostol, she was diagnosed as failed induction and oxytocin infusion was used. All data were gathered prospectively and recorded. Maternal demographic characteristics (maternal age, gestational age, parity, mode of delivery, first Bishop Score, neonatal Apgar score) were recorded for two groups and then were compared. The indication for induction and important outcomes of labor were recorded for each patient. During intervention, each patient was assessed for possible outcomes. Tachysystole was defined as the presence of at least five uterine contractions in two consecutive 10-min periods. Hyperstimulation syndrome was defined as tachysystole and/or hypertonus on cardiotocography, with fetal heart rate (FHR) alterations such as bradycardia (FHR < b110 bpm), late decelerations, and or loss fetal heart rate variability. 

*Outcomes Assay*

The primary outcome measure was the frequency of successful induction, defined as vaginal delivery within 24 h from the start of induction. Secondary outcomes included the rates of C/S due to fetal distress, time from first dose to active labor, induction-to delivery interval, duration of labor; number of misoprostol doses administered, and need for augmentation of labor with oxytocin. Neonatal outcomes included fetal heart rate (FHR) variation during labor, intrapartum meconium passage, intrapartum fetal death, and admission to the neonatal intensive care unit (NICU).

*Statistical analysis*

Analysis was performed with SPSS software (SPSS, Inc., Chicago, IL version 11.5), and then compared with χ² test and Exact Fisher test for comparison of qualitative data. After check of normality, Mann-Whitney U test and Kruskal-Wallis test were used, if normality not fitted, Independent t-test and ANOVA test used if normality fitted to data. p-value less 0.05 was considered statistically significant.

## Results and Discussion

A total of 140 women who admitted for termination of pregnancy were selected during the study procedure, 50 individuals received vaginal misoprostol and 90 cases attributed to the sublingual misoprostol group. 

There was no significant difference in the demographic characteristics between two studied groups of women (Table 1). However the Bishop Score in the sublingual group was significantly higher than the vaginal group (P = 0.021). 

**Table 1:** Demographic characteristics of patients in both groups

**p-value**	**Vaginal Group**	**Sublingual Group**	**Item**
0.67	24.34±4.05	24.65±4.42	Maternal Age( year)
0.20	1.36±.63	1.53±.86	Parity
			Indication for Induction
0.26	21(%42)	40(%43.5)	Post term
0.82	5(%10)	6(%6.5)	Oligihydraminios
0.15	18(%36)	29(%31.5)	PROM
0.66	1(%2)	1(%1.1)	Mild Preeclampsia
0.05	2 (%4)	0(%0)	Mild HTN
0.34	18(%36)	15(%17)	Loss motion
0.74	39.80±1.49	39.70±1.88	Gestational Age (weeks)
0.021	2.72±1.32	3.47±1.68	Bishop score

The clinical outcomes of induction in both groups are summarized in Table 2. Our findings showed that there weren’t any statistically significant differences between the number of administered doses of misoprostol every four hours (P > 0.05). Although the frequency of two doses were significantly higher than the other group (P = 0.43). About C/S indication, most of individuals showed absence of active labor from the sublingual group. 

**Table 2:** Clinical outcomes of induction in both groups

**p-value**	**Vaginal Group**	**Sublingual Group**	**Item**
			**Number of doses**
0.22	25(%50)	41(%44.6)	One dose
0.43	17(%34)	25(%27.2)	Two dose
0.19	3(%6)	12(%13)	Three dose
0.79	4(%8)	8(%8.7)	Four dose
0.94	1(%2)	2(%2.2)	Five dose
0.13	0(%0)	4(%4.3)	Six dose`
			**Mode of delivery**
0.60	45(%90)	78(%84.8)	Vaginal
0.72	5(%10)	13(%14.1)	CS
1.00	0(%0)	1(%1.1)	Vaccum
			**Indication for CS**
0.53	2(%4)	4 (%4.3)	Fetal distress
0.21	1(%2)	6(%6.5)	Absence of active labor
1.00	0(%0)	0(%0)	Uterine over activity
0.43	2(%4)	3(%3.3)	Failure to progress

Table 3 compared the time and outcome delivery in vaginal and sublingual misoprostol group. There weren’t any significant difference in passive and active phase and total time of delivery in two groups (P > 0.05). The maternal complication presentations included residue of placenta, tachysystole, vomiting, atoni and abdominal pain. Although there weren’t any significant differences in two studied group (P > 0.05) but abdominal pain and vomiting were showed more in sublingual group than vaginal group. Also, the neonatal complications including meconial and prolaps weren’t different between two studied groups (P > 0.05). 

**Table 3:** Compare time and outcome in vaginal and sublingual misoprostol in delivery

**p-value**	**Vaginal Group**	**Sublingual Group**	**Variables**
0.82	8.58±5.18	8.81±6.07	Passive phase of Delivery ( Hours)
0.95	2.87±1.72	2.89±1.90	Active phase of Delivery ( Hours)
0.61	11.08±3.41	11.62±6.76	Total time of Delivery ( Hours)
			**Maternal Complication**
0.87	1(%2)	2(%2.2)	Residue of Placenta
0.65	1(%2)	2(%2.2)	Tachysystole
0.34	5(%10)	11(%12)	Vomiting
0.09	3(%6)	3(%3.3)	Atoni
0.23	1(%2)	5(%5.5)	Abdominal Pain
			**Neonatal complication**
*1.00*	3(%6)	5(%5.5)	Meconial
*0.46*	0(%0)	1(%1.1)	prolaps

The results of the present study showed that sublingual misoprostol is effective as vaginal misoprostol for induction of labor with live term fetuses. 

Previous studies had already promised the beneficial effects of pharmacological over mechanical cervical ripening (20, 21). Previous studies showed that Misoprostol is an effective agent by oral, vaginal and sublingual administration for induction of labor before surgical termination of pregnancy. In our study, the vaginal and sublingual misoprostol weren’t associated with significant differences in the number of women in aspect of clinical outcomes and the maternal and neonatal complication. A randomized controlled trial evaluated the vaginal and sublingual misoprostol for second trimester abortion (22). They showed that a higher effectiveness of sublingual administration, but fever was more common in vaginal consumption (22). We didn’t evaluate these two methods in second trimester, but we found no significant difference among the maternal complication in studied period. Saxena *et al*. evaluated the sublingual versus vaginal routes of misoprostol in the first trimester abortions. They concluded that the sublingual form is more effective and convenient than vaginal forms for cervical dilatation (23). 

Different routes of misoprostol have been administrated for cervical priming (24-28). Both oral and vaginal forms seem to be equally effective (29). However, some women found the vaginal forms inconvenient and unacceptable (30). Tang *et al*. (2004) compared sublingual and vaginal misoprostol for preoperative cervical priming, prior to surgical termination and found similar preoperative side-effects within groups (31). However, sublingual misoprostol has the advantages like being more convenient to administer. We found both sublingual and vaginal misoprostol similarly effective in cervical priming. 

Zahran *et al*. (2009) like our study, evaluated sublingual versus vaginal misoprostol for induction of labor at term, but in randomized prospective placebo controlled study (32). We found similar results like Zahran *et al* study as fetal distress in both group had the highest frequency, but there was no difference between groups in case of induction to delivery interval, duration of labor, neonatal outcome or maternal side effects. However in comparison to their study (32), we found low number of attributed labors that had meconium staining in both studied groups, but less active labor in sublingual group.

They found sublingual route promised to higher patient’s satisfaction level (31). Although we didn’t evaluate the patient satisfaction level but our results, confirmed the previous studies results in other criterion.

## Conclusion

We concluded that sublingual misoprostol seems as effective as vaginal misoprostol for induction of labor at term. Furthermore misoprostol sublingual routes poses some advantages like convenient to administer and might be more suitable than vaginal form. 
